# Efficacy of perampanel by etiology in Japanese patients with epilepsy—subpopulation analysis of a prospective post‐marketing observational study

**DOI:** 10.1002/epi4.13002

**Published:** 2024-07-04

**Authors:** Miku Nakai, Shohei Nishimoto, Yoichi Higashibeppu, Yushi Inoue

**Affiliations:** ^1^ Neurology Department Medical Headquarters, Eisai Co., Ltd. Tokyo Japan; ^2^ Clinical Planning and Development Department Medical Headquarters, Eisai Co., Ltd. Tokyo Japan; ^3^ National Epilepsy Center NHO Shizuoka Institute of Epilepsy and Neurological Disorders Shizuoka Japan

**Keywords:** brain tumor, epilepsy, etiology, perampanel, stroke, trauma

## Abstract

**Objective:**

To examine the efficacy and safety of perampanel (PER) in patients with post‐stroke epilepsy (PSE), brain tumor‐related epilepsy (BTRE), and post‐traumatic epilepsy (PTE) using Japanese real‐world data.

**Methods:**

The prospective post‐marketing observational study included patients with focal seizures with or without focal to bilateral tonic–clonic seizures who received PER combination therapy. The observation period was 24 or 52 weeks after the initial PER administration. The safety and efficacy analysis included 3716 and 3272 patients, respectively. This post hoc analysis examined responder rate (50% reduction in seizure frequency), seizure‐free rate (proportion of patients who achieved seizure‐free), and safety in patients included in the post‐marketing study who had PSE, BTRE, and PTE in the 4 weeks prior to the last observation.

**Results:**

Overall, 402, 272, and 186 patients were included in the PSE, BTRE, and PTE subpopulations, and 2867 controls in the “Other” population (etiologies other than PSE, BTRE, or PTE). Mean modal dose (the most frequently administered dose) values at 52 weeks were 3.38, 3.36, 3.64, and 4.04 mg/day for PSE, BTRE, PTE, and “Other,” respectively; PER retention rates were 56.2%, 54.0%, 52.6%, and 59.7%, respectively. Responder rates (% [95% confidence interval]) were 82% (76.3%–86.5%), 78% (70.8%–83.7%), 67% (56.8%–75.6%), and 50% (47.9%–52.7%) for PSE, BTRE, PTE, and “Other,” respectively, and seizure‐free rates were 71% (64.5%–76.5%), 62% (54.1%–69.0%), 50% (40.6%–60.4%), and 28% (25.8%–30.1%), respectively. Adverse drug reactions tended to occur less frequently in the PSE (14.7%), BTRE (16.5%), and PTE (16.7%) subpopulations than in the “Other” population (26.3%).

**Significance:**

In real‐world clinical conditions, efficacy and tolerability for PER combination therapy were observed at low PER doses for the PSE, BTRE, and PTE subpopulations.

**Plain Language Summary:**

To find out how well the medication perampanel works and whether it is safe for people who have epilepsy after having had a stroke, brain tumor, or head injury, we used information from real‐life medical situations in Japan. We looked at the data of about 3700 Japanese patients with epilepsy who were treated with perampanel. We found that perampanel was used at lower doses and better at controlling seizures, and had fewer side effects for patients with epilepsy caused by these etiologies than the control group.

**Key points**

- Perampanel showed good efficacy and safety for epilepsy due to cerebrovascular disorders (PSE), brain tumors (BTRE), or head trauma (PTE).
- Perampanel doses were lower in the PSE, BTRE, and PTE subpopulations compared to the “Other” group (epilepsy due to other etiologies).
- Perampanel resulted in higher responder and seizure‐free rates in the PSE, BTRE, and PTE subpopulations than in the “Other” group.
- The efficacy of perampanel by seizure type was consistently higher in the PSE, BTRE, and PTE subpopulations compared to the “Other” group.
- The incidence of overall ADRs and nervous system disorders was low in the PSE, BTRE, and PTE subpopulations.

## 1 INTRODUCTION

With the recent population aging, the incidence of epilepsy in the elderly has increased, especially among those aged over 60 or 65 years.^1^ Major causes of new‐onset epilepsy in the elderly include post‐stroke epilepsy (PSE) in those with cerebrovascular disease, epilepsy related to neurodegenerative disorders, brain tumor‐related epilepsy (BTRE), and post‐traumatic epilepsy (PTE).^1^, ^2^ The most important risk factors for new‐onset epilepsy in the elderly are stroke and other cerebrovascular diseases, representing 30%–50% of all identified causes.^2^ PSE is reported to occur in about 6%–18% of stroke patients.^3^ According to two review articles, 35%–70% of patients with brain tumors develop BTRE.^4^, ^5^ PTE accounts for 5% of all epilepsy patients referred to specialized epilepsy centers.^6^

Anti‐seizure medications (ASMs) are used to treat these forms of epilepsy, but there are currently no established guidelines for drug therapy due to a lack of evidence from randomized controlled trials. However, newer generation ASMs have been suggested to be better tolerated and safer than older generation ASMs, and clinicians are now considering the use of novel ASMs in epilepsy.^7^, ^8^ Perampanel (PER) is a highly selective and non‐competitive postsynaptic α‐amino‐3‐hydroxy‐5‐methyl‐4‐isoxazolepropionic acid (AMPA) receptor antagonist. To date, PER is available in tablet and granule form in Japan, and an infusion formulation was approved for manufacture and sale in January 2024.^9^

In 2016, a prospective post‐marketing observational study of the efficacy and safety of PER in patients with epilepsy aged ≥18 years was initiated in Japan.^10^ The study included 3716 patients in the safety analysis and 3272 in the efficacy analysis. Analysis of the rate of achievement of 50% reduction in seizure frequency (responder rate) by patient baseline characteristics revealed that brain tumors (odds ratio [OR] 2.102) and cerebrovascular disease (OR 2.086) were factors that affected the efficacy of PER treatment.^10^ In addition, multivariate analysis of overall improvement by patient baseline characteristics showed that head injury (OR 1.616) and cerebrovascular disease (OR 1.785) were also factors affecting efficacy (data on file).

These results suggest that PSE, BTRE, and PTE are factors that have an effect on PER efficacy.^10^ Furthermore, an association between the etiology of these types of epilepsy and AMPA receptors has also been suggested.^11^, ^12^, ^13^ Currently, there is a lack of clinical evidence for PER use, particularly in patients with PSE and PTE. Therefore, we performed a post hoc subpopulation analysis using data from a prospective Japanese post‐marketing observational study^10^ to obtain detailed information on the efficacy and safety of PER in the three etiologic populations of PSE, BTRE, and PTE.

## 2 METHODS

### 2.1 Study design

This is a post hoc subpopulation analysis of a prospective post‐marketing observational study conducted in Japan. The study design of the prospective post‐marketing observational study was published previously.^10^ The study adhered to Good Post‐marketing Study Practice (GPSP) and was registered at Clinicaltrials.gov under the identifier NCT03059329. Because GPSP does not mandate patient consent, it was not required for our study. Patient registration was conducted via a central registration system (Electronic Data Capture system). Before this post hoc subpopulation analysis, the data were anonymized per Eisai's Regulations for Handling Anonymized Processed Information to protect the patients' personal information.

As this was an observational and non‐interventional study, drugs were prescribed by the treating physicians. Per the package insert,^14^ the initial oral dosage of PER for patients aged 12 years or older was 2 mg once daily at bedtime, which could be increased by 2 mg at ≥1‐week intervals. The maintenance dose of PER was 4–8 mg once daily in the absence of concomitant ASMs that could accelerate PER metabolism or 8–12 mg once daily in the presence of such concomitant drugs. Of note, when the study was conducted, PER monotherapy was not approved in Japan; thus, the efficacy and safety of PER as combination therapy were evaluated.

The observation period included 52 weeks after the first dose. Once 52 weeks of data had been collected for 300 patients, only the first 24 weeks of data were analyzed for patients with an observation period of less than 52 weeks. The last‐observation‐carried‐forward approach was used for missing data at the last efficacy assessment. To better reflect real‐world clinical practice, there were no limitations on changes in doses of concomitant ASMs or adding new medications to the regimen.

### 2.2 Patients

Patients included in the study were aged 18 years or older and had focal seizures (FS) with or without focal to bilateral tonic–clonic seizures (FBTCS) or generalized tonic–clonic seizures (GTCS) according to the 2017 International League Against Epilepsy classification.^15^ The PER post‐marketing observational study evaluated 3769 patients. This post hoc subpopulation analysis evaluated patients with PSE, BTRE, PTE, and those in the “Other” population who had etiologies other than PSE, BTRE, or PTE.

### 2.3 Data collection

Data collected included sex, age, weight, clinical characteristics, epilepsy classification, disease duration, patient background such as comorbidities, and data related to PER, including daily dose, retention rate, and reason for withdrawal. The daily dose was defined as the most frequently administered dose (i.e., modal dose) for each patient during the study. If multiple doses were used, then the higher dose was considered the modal dose.

### 2.4 Study outcomes

To assess the safety of PER, adverse drug reactions (ADRs) potentially related to PER were monitored using MedDRA version 23.0. Current seizures and seizure‐related events were not considered ADRs. The effectiveness of PER was assessed by comparing the seizure frequency during the 4 weeks immediately before the last observation with the seizure frequency at baseline.

For focal aware seizures (FAS) with/without motor signs, focal onset impaired awareness seizures (FIAS), and FBTCS, the responder rate (the percentage of patients who achieved a 50% or greater reduction in seizure frequency in the 4 weeks prior to last observation) and the seizure‐free rate (the percentage of patients who were seizure‐free [i.e., 100% reduction in seizure frequency] in the 4 weeks prior to last observation) were evaluated for each type of seizure. Overall improvement was also assessed on a 7‐point scale, with 1 point representing markedly worse and 7 points representing markedly improved; cases with marked improvement (7 points), considerable improvement (6 points), and slight improvement (5 points) were considered “improved” and subjected to an improvement rate analysis. Improvement rate was defined as the percentage of patients who were considered “improved” and was calculated using the number of “improved” patients as the numerator and the number of patients in the efficacy analysis set (excluding patients who could not be evaluated) as the denominator.

### 2.5 Statistical analysis

The retention rate of PER was estimated using the Kaplan–Meier method. The responder and seizure‐free rates were calculated for each type of seizure. Descriptive statistics, including the calculation of proportions and their confidence intervals (CIs), were used to summarize the efficacy data. The 95% CIs for each condition were calculated using the Clopper‐Pearson method to account for the binomial nature of the data.

Due to the exploratory nature of this analysis and the lack of a priori‐defined hypothesis, a multiplicity correction was intentionally not applied. The analysis focused on identifying patterns and trends rather than making formal statistical inferences about differences between conditions. All statistical analyses were performed using R version 4.3.0 (R Foundation for Statistical Computing, Vienna, Austria) and the “binom” package for CI calculations.

## 3 RESULTS

### 3.1 Patient demographic and baseline characteristics

The PER post‐marketing observational study evaluated 3769 patients. Of those, patients with PSE (*n* = 402), BTRE (*n* = 272), PTE (*n* = 186), and types of epilepsy other than PSE, BTRE, or PTE (“Other”; *n* = 2867) were evaluated in this post hoc subpopulation analysis. Table 1 summarizes the baseline characteristics of patients in the PSE, BTRE, and PTE subpopulations and the “Other” population. The proportion of patients aged ≥65 years was higher in the PSE, BTRE, and PTE subpopulations compared with the “Other” population (32%–55% vs 11.2%) and the proportion of outpatients was lower (51.5%–59.9% vs 86.8%). The proportion of patients who had a disease duration of ≥10 years was less than 50% in the PSE, BTRE, and PTE subpopulations, which was lower than approximately 80% in the “Other” population. In the PSE, BTRE, and PTE subpopulations and the “Other” population, most patients were taking concomitant ASMs at baseline. The proportion of patients taking one concomitant ASM was 64.2%, 57.0%, and 50.8% in the PSE, BTRE, and PTE subpopulations, respectively, and was lower in the “Other” population, with an equal distribution of approximately 25% of patients taking one, two, or three concomitant ASMs. The PSE and BTRE subpopulations had lower rates of comorbid psychiatric symptoms (aggression, depression, other) within 2 years prior to study entry than the “Other” population, while the PTE subpopulation had similar rates of aggression and depression compared with the “Other” population.

**TABLE 1:** Baseline patient characteristics by epilepsy etiology subpopulation.

Characteristics	Epilepsy etiology			Other *n* = 2867
	PSE *n* = 402	BTRE *n* = 272	PTE *n* = 186	
Male	247 (61.4)	144 (52.9)	128 (68.8)	1453 (50.7)
Female	155 (38.6)	128 (47.1)	58 (31.2)	1414 (49.3)
Age (years)				
≥18 to <40	54 (13.4)	53 (19.5)	44 (23.7)	1514 (52.8)
≥40 to <65	127 (31.6)	132 (48.5)	73 (39.2)	1033 (36.0)
≥65	221 (55.0)	87 (32.0)	69 (37.1)	320 (11.2)
Body weight (kg)				
<50	91 (22.6)	51 (18.8)	37 (19.9)	571 (19.9)
≥50 to <80	195 (48.5)	142 (52.2)	100 (53.8)	1057 (36.9)
≥80	22 (5.5)	14 (5.1)	4 (2.2)	178 (6.2)
Unknown	94 (23.4)	65 (23.9)	45 (24.2)	1061 (37.0)
Outpatients/inpatients				
Outpatient	207 (51.5)	163 (59.9)	110 (59.1)	2488 (86.8)
Inpatient	195 (48.5)	109 (40.1)	76 (40.9)	379 (13.2)
Disease duration (years)				
<10	283 (70.4)	175 (64.3)	90 (48.4)	632 (22.0)
≥10	106 (26.4)	87 (32.0)	90 (48.4)	2156 (75.2)
Unknown	13 (3.2)	10 (3.7)	6 (3.2)	79 (2.8)
Focus localization				
Frontal lobe	128 (31.8)	112 (41.2)	71 (38.2)	463 (16.1)
Temporal lobe	93 (23.1)	77 (28.3)	42 (22.6)	814 (28.4)
Parietal lobe	61 (15.2)	53 (19.5)	12 (6.5)	79 (2.8)
Occipital lobe	17 (4.2)	11 (4.0)	5 (2.7)	77 (2.7)
Multilobed	21 (5.2)	13 (4.8)	12 (6.5)	165 (5.8)
Use of concomitant ASMs at baseline	385 (95.8)	263 (96.7)	177 (95.2)	2840 (99.1)
Number of concomitant ASMs				
1	247 (64.2)	150 (57.0)	90 (50.8)	707 (24.9)
2	89 (23.1)	80 (30.4)	47 (26.6)	737 (26.0)
3	28 (7.3)	25 (9.5)	22 (12.4)	743 (26.2)
≥4	21 (5.5)	8 (3.0)	18 (10.2)	653 (23.0)
Psychiatric comorbidity within 2 years before study				
No/Unknown	364 (90.5)	258 (94.9)	163 (87.6)	2344 (81.8)
Yes	38 (9.5)	14 (5.1)	23 (12.4)	523 (18.2)
Aggression	16 (4.0)	4 (1.5)	14 (7.5)	190 (6.6)
Depression	16 (4.0)	7 (2.6)	10 (5.4)	171 (6.0)
Other	10 (2.5)	4 (1.5)	5 (2.7)	250 (8.7)

*Note*: Data are presented as *n* (%).

Abbreviations: ASM, anti‐seizure medication; BTRE, brain tumor‐related epilepsy; PSE, post‐stroke epilepsy; PTE, post‐traumatic epilepsy; SD, standard deviation.

### 3.2 Perampanel modal dose and retention rate

The doses of PER for the PSE, BTRE, and PTE subpopulations and the “Other” population are shown in Table 2. In the three epilepsy subpopulations, the mean values of modal dose (3.38, 3.36, and 3.64 mg/day, respectively) and maximum dose (3.82, 3.87, and 4.15 mg/day, respectively) were lower than in the “Other” population (4.04 and 4.93 mg/day, respectively).

**TABLE 2:** Dose of perampanel by epilepsy etiology subpopulation.

Dose^a^	Epilepsy etiology			Other *n* = 2867
	PSE *n* = 402	BTRE *n* = 272	PTE *n* = 186	
Initial dose	2.00	1.99	1.97	1.90
Modal dose	3.38	3.36	3.64	4.04
Maximum dose	3.82	3.87	4.15	4.93

*Note*: The modal dose was defined as the most frequently administrated dose.

Abbreviations: BTRE, brain tumor‐related epilepsy; PSE, post‐stroke epilepsy; PTE, post‐traumatic epilepsy.

^a^ Mean dose of perampanel (mg/day).

In the distribution of patients by modal dose, 2 mg/day was most common in the PSE and BTRE subpopulations (>50%), followed by 4 mg/day. Patients with PTE and those in the “Other” population were primarily treated with doses between 2 and 4 mg/day (Table S1).

**FIGURE 1:**
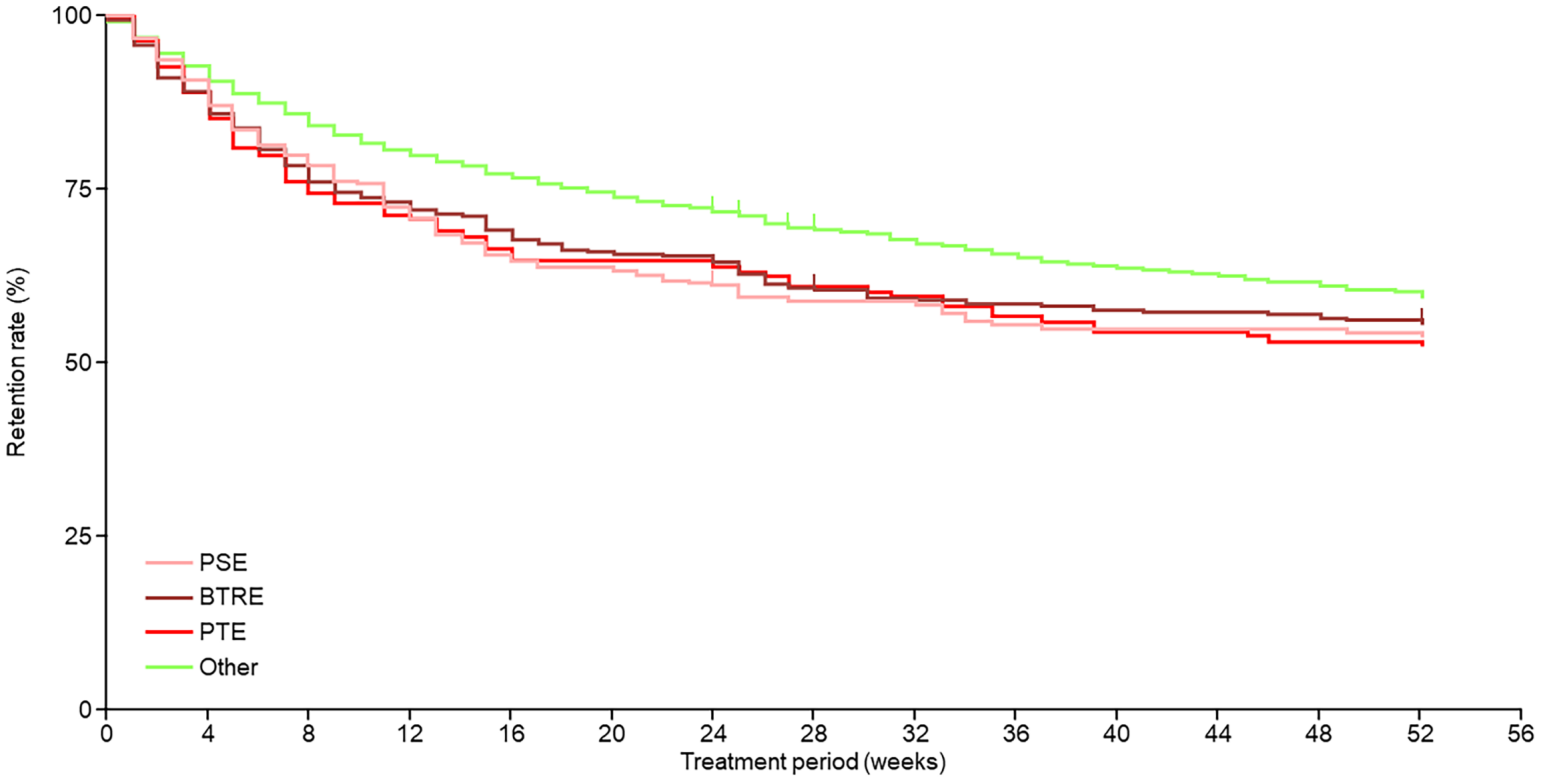
Retention rate of perampanel. BTRE, brain tumor‐related epilepsy; PSE, post‐stroke epilepsy; PTE, post‐traumatic epilepsy. The “Other” population consisted of patients with epilepsy of causes other than PSE, BTRE, and PTE.

The respective PER retention rates in the PSE, BTRE, and PTE subpopulations and the “Other” population were 64.9%, 61.4%, 64.0%, and 72.2% at 6 months, and 56.2%, 54.0%, 52.6%, and 59.7% at 12 months (Figure 1). Retention rates tended to be lower in the PSE, BTRE, and PTE subpopulations than in the “Other” population.

The most common reason for the discontinuation of PER in the PSE, BTRE, and PTE subpopulations and in the “Other” population was “adverse events” (16.9%, 23.5%, 16.1%, and 18.9%, respectively), followed by “did not come to the hospital within 24 weeks of treatment” (11.4%, 9.6%, 12.9%, and 4.1%, respectively), which was reported more frequently by patients in the PSE, BTRE, and PTE subpopulations than in the “Other” population.

### 3.3 Efficacy outcomes

**FIGURE 2:**
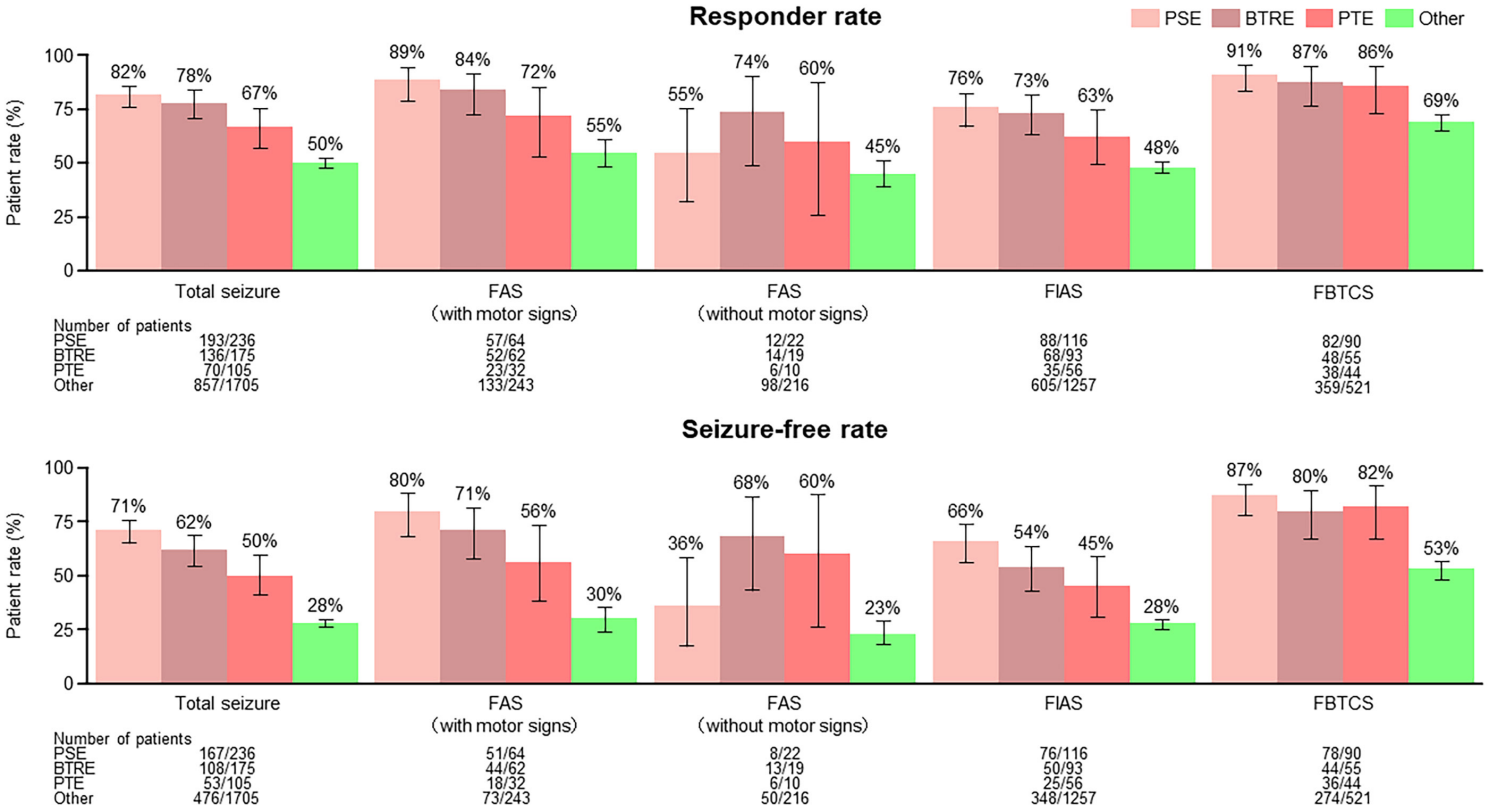
Efficacy of perampanel for PSE, BTRE, PTE, and “Other” populations. BTRE, brain tumor‐related epilepsy; FAS, focal aware seizure; FBTCS, focal to bilateral tonic–clonic seizure; FIAS, focal onset impaired awareness seizure; PSE, post‐stroke epilepsy; PTE, post‐traumatic epilepsy. The “Other” population consisted of patients with epilepsy of causes other than PSE, BTRE, and PTE. Error bars are presented as 95% CIs. CI, confidence interval.

Regarding the effect of PER on total seizure in the PSE, BTRE, and PTE subpopulations and the “Other” population during the 52‐week observation period after the first PER administration, the responder rate (% [95% CI]) was 82% (76.3%–86.5%), 78 (70.8%–83.7%), 67% (56.8%–75.6%), and 50% (47.9%–52.7%), respectively, and the seizure‐free rate was 71% (64.5%–76.5%), 62% (54.1%–69.0%), 50% (40.6%–60.4%), and 28% (25.8%–30.1%), respectively. The efficacy of PER for seizure control was numerically higher in the PSE, BTRE, and PTE subpopulations than in the “Other” population (Figure 2). The effects of PER by seizure type also showed a trend toward higher effectiveness in the PSE, BTRE, and PTE subpopulations than in the “Other” population for all seizure types.

The overall improvement rate (% [95% CI]) assessed by physicians also tended to be higher in the PSE (74.0% [68.9%–78.7%]), BTRE (66% [60.0%–72.4%]), and PTE (66% [57.4%–73.1%]) subpopulations than in the “Other” population (56% [54.5%–58.4%]) (Figure S1).

**FIGURE 3:**
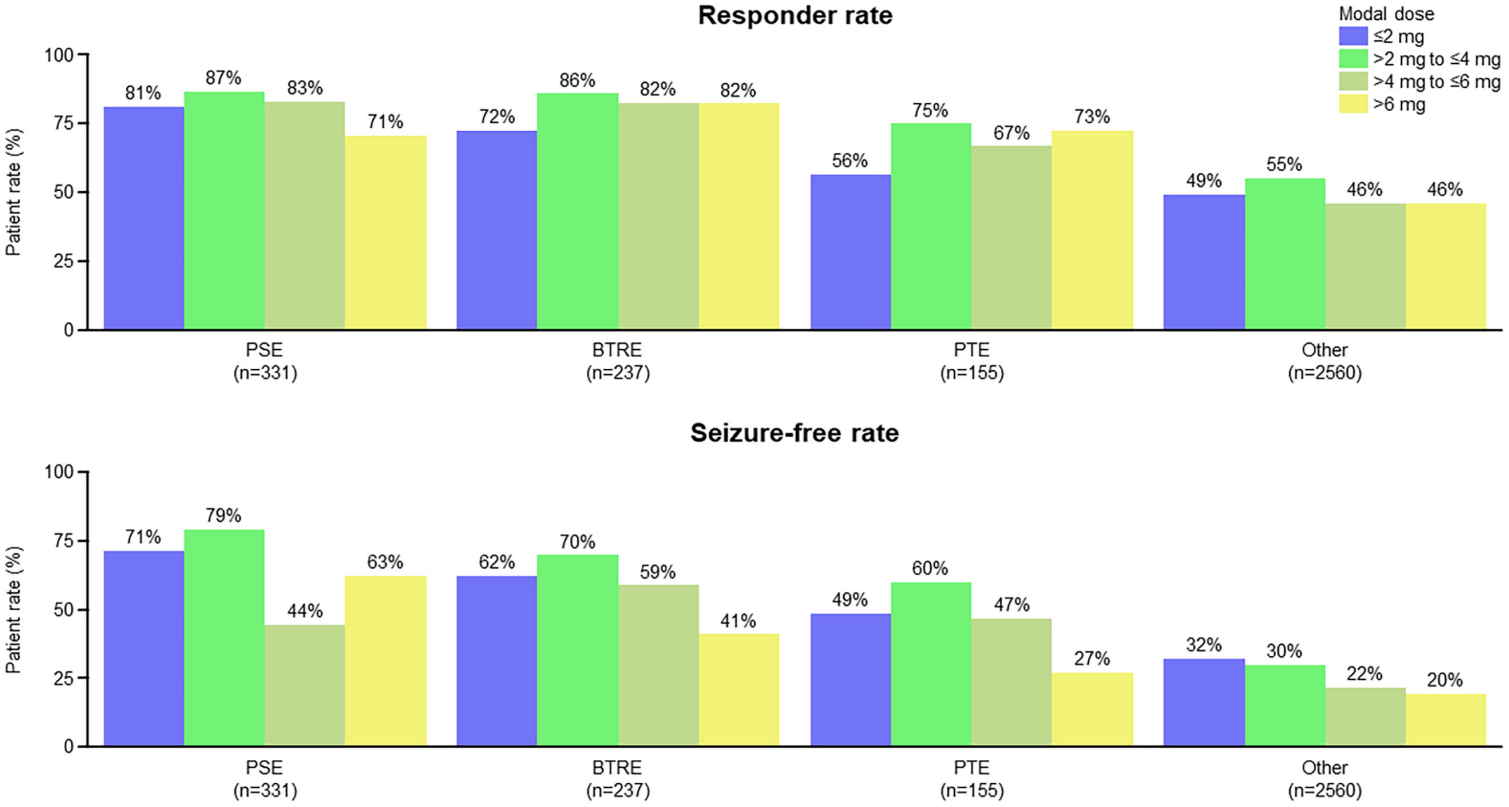
Efficacy of perampanel by modal dose. BTRE, brain tumor‐related epilepsy; PSE, post‐stroke epilepsy; PTE, post‐traumatic epilepsy. The “Other” population consisted of patients with epilepsy of causes other than PSE, BTRE, and PTE.

Regarding the efficacy of PER by modal dose, both responder and seizure‐free rates were found to be dose‐independent and effective in all populations (PSE, BTRE, PTE, and “Other”) (Figure 3). Most patients with PER modal dose ≤2 mg/day remained at the 2 mg/day dose (Table S1).

### 3.4 Safety outcomes

The incidence of ADRs per subpopulation is summarized in Table 3. The frequency of overall ADRs in the PSE (14.7%), BTRE (16.5%), and PTE (16.7%) subpopulations showed a trend toward a lower frequency of ADR occurrence compared with the “Other” population (26.3%). The incidence of psychiatric and nervous system disorders in the PSE, BTRE, and PTE subpopulations was also lower than in the “Other” population.

**TABLE 3:** Incidence of ADRs.

ADRs	Epilepsy etiology			Other *n* = 2867
	PSE *n* = 402	BTRE *n* = 272	PTE *n* = 186	
Overall	59 (14.7)	45 (16.5)	31 (16.7)	755 (26.3)
Psychiatric disorders	26 (6.5)	15 (5.5)	11 (5.9)	248 (8.7)
Irritability	9 (2.2)	3 (1.1)	5 (2.7)	134 (4.7)
Aggression	6 (1.5)	–	3 (1.6)	54 (1.9)
Agitation	6 (1.5)	–	–	48 (1.7)
Anger	6 (1.5)	10 (3.7)	3 (1.6)	32 (1.1)
Nervous system disorders	38 (9.5)	32 (11.8)	22 (11.8)	552 (19.3)
Somnolence	27 (6.7)	19 (7.0)	11 (5.9)	333 (11.6)
Dizziness	15 (3.7)	14 (5.1)	13 (7.0)	289 (10.1)

*Note*: Data are presented as *n* (%). ADRs of more than 1% are listed.

Abbreviations: ADR, adverse drug reactions; BTRE, brain tumor‐related epilepsy; PSE, post‐stroke epilepsy; PTE, post‐traumatic epilepsy.

## 4 DISCUSSION

### 4.1 Summary of the main findings of the post hoc subpopulation analysis

The three subpopulations analyzed in our study, PSE, BTRE, and PTE, achieved promising efficacy and safety consistent with those of the control population (i.e., the “Other” population). In the three subpopulations, there was a trend toward lower doses and higher responder and seizure‐free rates compared with the control population. The overall improvement rate was higher in the three subpopulations than the control population, with the same trend shown in the responder rate and seizure‐free rate for total seizure.

It is possible that the higher effectiveness observed in the three subpopulations relative to the control population was attributable to differences in patient baseline characteristics. Many of the patients in these three subpopulations had fewer concomitant ASMs and may represent a population of patients who were treated with PER at an earlier stage at relatively early stage of their epilepsy.

Of note, the modal dose in the three subgroups was less than the recommended dose of PER, which is 4–8 mg/day (up to 12 mg/day with concomitant enzyme‐inducing ASMs [EIASMs]), and previous clinical trials were based on this dose.^16^ Also, in our study, more than 40% of patients in the “Other” group received less than 4 mg/day of PER (Table S1). These findings suggest that PER is often prescribed below the recommended dose in real clinical practice. It has also been reported that titration of PER occurs more slowly in real‐world conditions than in clinical trials.^17^ Sufficient therapeutic effect was achieved during the slow dose escalation process, possibly leading primary physicians to set the maintenance dose before the recommended dose was reached (Table 2, Figure 3). This may explain why the modal dose in the PSE, BTRE, and PTE subgroups in this analysis was less than the recommended dose of 4 mg/day.

The higher treatment discontinuation rate in the three subpopulations versus the control population was thought to be due to a higher proportion of patients who were prescribed PER upon admission and then transferred to the hospital upon discharge or for other reasons, resulting in a higher proportion of patients who did not go to the hospital during the treatment period. This inference is consistent with the high number of dropouts within 24 weeks.

The incidences of both overall ADRs and nervous system disorders were lower in the three subpopulations than in the control population, which may be attributed to the lower modal and maximum doses of PER in the subpopulations. The incidence of psychiatric disorders was also lower in the three subpopulations. This may have been influenced by the lower rate of psychiatric comorbidities at baseline in the subpopulations.

### 4.2 Post‐stroke epilepsy

Because there is little validation of the efficacy of ASM in patients with PSE,^3^ no clear guidelines have been proposed for the selection of ASM for PSE,^18^ including in Japan. Because of the anticipated concomitant use of anticoagulants and lifestyle medications in stroke, newer‐generation ASMs, generally considered to have fewer drug–drug interactions, are recommended.^19^

During the acute phase of stroke, glutamate levels in the brain rise and increase tissue injury.^20^ Glutamate is involved in the development of seizures and epilepsy.^21^ Additionally, the expression of calcium‐permeable AMPA receptors is increased in PSE.^20^ As PER directly inhibits AMPA receptors, it could be a viable therapeutic option for PSE.^21^ Nonclinical animal models have shown that PER may protect the brain from glutamate toxicity.^22^, ^23^, ^24^, ^25^, ^26^, ^27^

Clinical results from a pooled analysis of 100 patients with epilepsy associated with cerebrovascular disease showed a responder rate of 70.6% in focal‐onset seizures and 66.7% in FBTCS at 6 months.^28^ In a multi‐center study of 28 PSE patients treated with perampanel, a complete seizure‐free rate at 6 months of 80% was reported, and favorable outcomes were observed in patients with FBTCS in particular.^29^ Although not directly comparable, a study of lacosamide add‐on therapy in patients with cerebrovascular epilepsy etiology reported a responder rate of 56.7%.^30^

The results of our study in PSE are consistent with the results of these studies, with a responder rate of 82% and a seizure‐free rate of 71%. This suggests that PER may be an effective adjunctive treatment option for PSE.

### 4.3 Brain tumor‐related epilepsy

In BTRE, there are no established drugs recommended by current treatment guidelines. A recent systematic review,^31^ as well as other studies^32^ have proposed the use of levetiracetam or lamotrigine monotherapy as first‐line options for patients with primary or metastatic brain tumors with seizures. Vacher et al recommended carbamazepine, lacosamide, oxcarbazepine, PER, sodium valproate, topiramate, and zonisamide as second line options.^32^ However, they noted that non‐EIASMs should be preferentially recommended over EIASMs, such as carbamazepine and phenytoin, for BTRE because of concerns about drug–drug interactions with chemotherapeutic agents and other drugs in the treatment of brain tumors.^32^

The glioma cell surface contains the cystine‐glutamate transporter (xCT), which releases the excitatory neurotransmitter glutamate.^12^ Decreased expression of the glutamate transporter EAAT2, which is highly expressed in astrocytes, leads to reduced glutamate uptake.^33^ Calcium‐permeable AMPA‐type receptors can significantly impact the growth and migration of malignant gliomas.^34^ Low‐grade epilepsy‐associated neuroepithelial tumors, gangliogliomas, and dysembryoplastic neuroepithelial tumors are typical examples. In these tumors, glutamatergic transmission seems to play a vital role in the pathogenesis of epilepsy.^35^, ^36^ Indeed, current nonclinical evidence on PER suggests that it has promising antitumor activity but no effect on tumor progression. As clinical data supporting these effects of PER are scarce, further studies are needed.^37^

Several small trials in patients with BTRE have been conducted to evaluate the efficacy and safety of PER combination therapy. A systematic review showed that PER combination therapy for BTRE resulted in responder rates of 75%–95% at 6 to 12 months, with retention rates ranging from 56% to 83%.^38^ The findings in patients with BTRE in the current study align with earlier studies. Although not directly comparable to our study, several studies of patients with BTRE treated with lacosamide as add‐on therapy have reported responder rates ranging from 66.3% to 86.4%.^39^, ^40^, ^41^

Our study reports a responder rate of 78% and a seizure‐free rate of 62%. These results indicate that PER is effective as a combination therapy for BTRE that is refractory to other treatments.

### 4.4 Post‐traumatic epilepsy

PTE is characterized by focal seizures and tonic–clonic seizures, with some reports indicating a high incidence of tonic–clonic seizures.^42^ Therefore, a drug with tonic–clonic seizure suppression may be desirable as an ASM.

Delayed onset seizures occur because of cortical damage caused by free radicals generated from extravascularly drained blood and increased excitability due to glutamate accumulation.^43^, ^44^ ASMs are expected to be used not only for their seizure‐preventive effects but also for their cerebroprotective properties,^44^ which were shown for PER in a head trauma rat model.^27^, ^45^

Thus far, no clinical trials of PER in PTE have been reported, but some real‐world data have shown that PER is effective in patient populations including PTE.^10^, ^46^, ^47^ A recent case study reported that adjunctive therapy with PER for super‐refractory status epilepticus due to PTE resulted in the patient being seizure free.^48^ Thus, PER can be considered an adjunctive therapy option in patients with PTE. The results of our study showed a responder rate of 67% in patients with PTE and a seizure‐free rate of 50%, supporting its effectiveness as adjunctive therapy in treatment‐resistant PTE.

## 5 LIMITATIONS

This study had some limitations. The seizure diaries were not used, therefore there may be a lack of rigorousness in the information of seizure occurrence. The evaluation was conducted under conditions wherein the dosage of the concomitant ASM could have been changed. Furthermore, detailed information on the causative conditions of PSE, BTRE, and PTE (such as degree or severity of conditions) was unknown. Furthermore, a significance test was not performed as this was an exploratory post hoc analysis. Finally, in the present study, we evaluated patients undergoing PER combination therapy; future evaluation of PER monotherapy will be needed.

## 6 CONCLUSIONS

This post hoc analysis of a Japanese post‐marketing study revealed that combination therapy with PER was more effective in reducing seizures and achieving seizure‐free in patients with epilepsy caused by PSE, BTRE, and PTE compared with the “Other” population. There were no major tolerability issues, and the safety of PER was confirmed.

These findings indicate that adjunctive treatment with PER may be beneficial for patients with epilepsy caused by brain tumors, cerebrovascular disorders, and head trauma. However, additional data are needed for in‐depth understanding of both PER monotherapy and combination therapy.

## AUTHOR CONTRIBUTIONS

Y. Inoue designed the study. All authors were involved in data analysis and interpretation, and writing and reviewing the manuscript. All authors approved the final version of the manuscript for submission.

## CONFLICT OF INTEREST STATEMENT

M. Nakai, S. Nishimoto, and Y. Higashibeppu are employees of Eisai Co., Ltd. Y. Inoue has received consultancy fees from Eisai Co., Ltd. and UCB Japan Co., Ltd. We confirm that we have read the Journal's position on issues involved in ethical publication and affirm that this report is consistent with those guidelines.

## Supporting information


Appendix S1.

## Data Availability

Due to privacy and ethical restrictions that were in place when the protocol was approved, raw data from this study are not available. Code used in the analysis and metadata generated in the study are available from the corresponding author upon reasonable request within the privacy policy of the participant informed consent.
