# Supplementary material for: Efficacy of perampanel by etiology in Japanese patients with epilepsy—subpopulation analysis of a prospective post‐marketing observational study
Source: Epilepsia Open. 2024 Jul 4;9(5):1772–82. doi: 10.1002/epi4.13002 (PMC11450607; doi:10.1002/epi4.13002)
Supplement: Supplementary file 1 — Appendix S1. [file EPI4-9-1772-s001.docx]

**Supporting information**

**Table S1**. Distribution of modal dose by epilepsy etiology subpopulation

| **Modal dose**  **(mg/day)** | **Epilepsy etiology** | | | **Other *n*=2560** |
| --- | --- | --- | --- | --- |
|  | **PSE *n*=331** | **BTRE *n*=237** | **PTE *n*=155** |  |
| <2 | 9 (2.7) | 5 (2.1) | 5 (3.2) | 116 (4.5) |
| 2 | 180 (54.4) | 131 (55.3) | 59 (38.1) | 929 (36.3) |
| >2 to <4 | 2 (0.6) | - | - | 52 (2.0) |
| 4 | 82 (24.8) | 57 (24.1) | 52 (33.6) | 741 (29.0) |
| > 4 to <6 | 1 (0.3) | - | 1 (0.7) | 21 (0.8) |
| 6 | 23 (7.0) | 23 (9.7) | 20 (12.9) | 302 (11.8) |
| >6 to <8 | - | - | - | 9 (0.4) |
| 8 | 30 (9.1) | 17 (7.2) | 16 (10.3) | 279 (10.9) |
| >8 | 4 (1.2) | 4 (1.7) | 2 (1.3) | 111 (4.3) |

Data are presented as *n* (%).

BTRE, brain tumor-related epilepsy; PSE, post-stroke epilepsy; PTE, post-traumatic epilepsy

The modal dose was defined as the most frequently administrated dose.

**Figure S1.** Overall improvement rate assessed by physicians


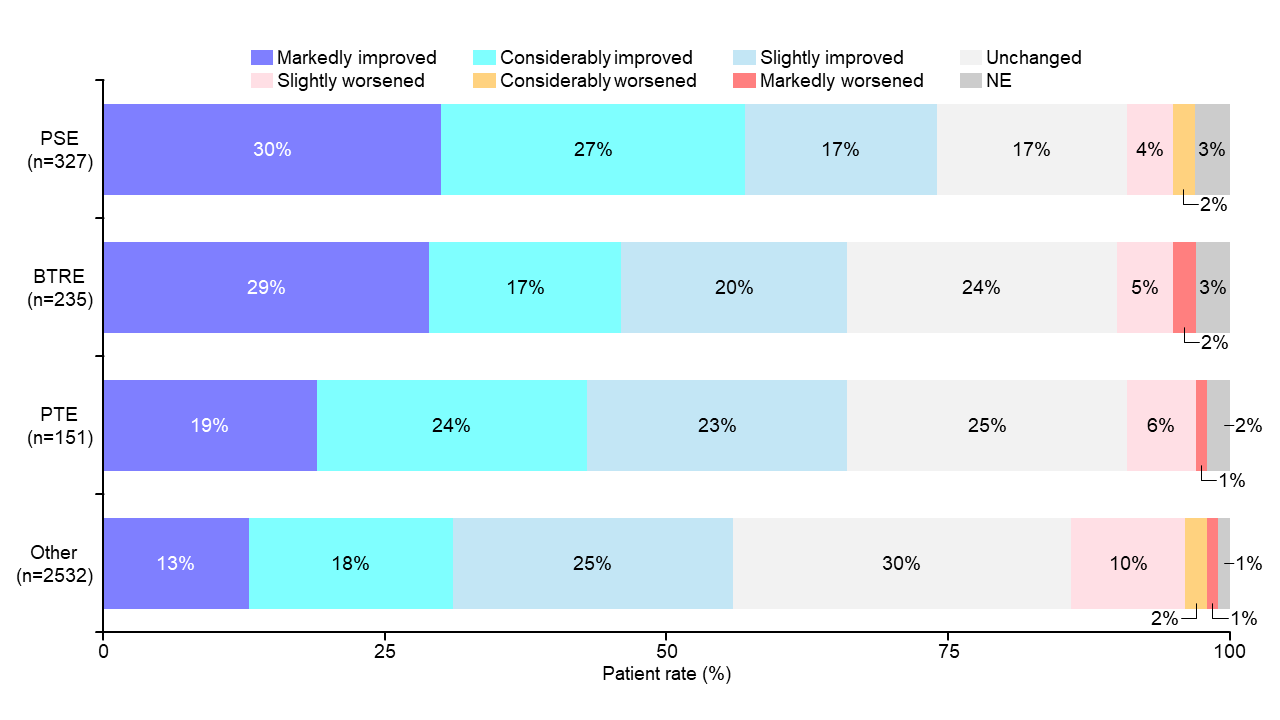


BTRE, brain tumor-related epilepsy; NE, not evaluable; PSE, post-stroke epilepsy; PTE, post-traumatic epilepsy
